# Mel Greaves: Cancer through the Lens of Evolution

**DOI:** 10.1016/j.trecan.2016.10.007

**Published:** 2016-10

**Figure:**
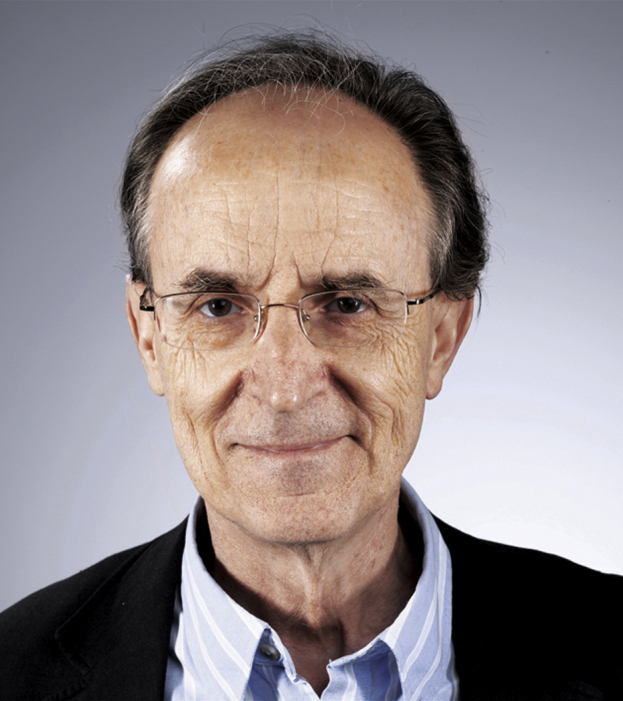

‘One general law, leading to the advancement of all organic beings, namely, multiply, vary, let the strongest live and the weakest die’ (Charles Darwin in *On the Origin of Species*). More than a century and a half after Darwin made selection a central biological concept, evolutionary biology is at the vanguard of cancer research. How is the Darwinian framework shedding light on cancer incidence, metastasis, or resistance and recurrence? Among the leaders answering these questions is Mel Greaves, Founding Director of the Centre for Evolution at The Institute of Cancer Research (ICR) in London, UK. His pioneering studies led to some of the most fascinating discoveries on the aetiology of childhood leukaemia. We asked Dr Greaves to give us his view on the implications and benefits of embracing an evolutionary perspective towards cancer biology and therapy.

## You have always advocated that cancer should be studied from an evolutionary perspective. Why is that so?

My answer is both pragmatic and philosophical. Cancer, like other complex problems in biomedicine and science generally, is more likely to be tackled successfully if we have a coherent and unifying view of its fundamental nature.

In the 19th century, cancer was widely regarded by surgeons and pathologists as a ‘disease of civilization or stress’. Not much of an advance on the ancient Greek view that cancer was a manifestation of a melancholic predisposition. In the 20th century, inspired by Boveri, it was confidently concluded that cancer was ‘a genetic disease’. This has now been superseded in the 21st century with the notion of cancer as ‘a disease of the genome’.

There is of course an element of truth in those latter descriptions, but in my view they do not cut it. They fall short and lack context. The problem here relates to how scientists of different disciplines handle the notion and meaning of ‘cause’. It is not surprising that epidemiologists, cell and molecular biologists, geneticists, and oncologists, at least in the past, saw the issue of cause somewhat differently with respect to questions and answers.

To my mind, any fundamental explanation of cancer as a disease has to accommodate three related challenges to be credible. One, what are the key ingredients of cancer risk? Two, what drives (or restrains) the emergence of a malignant tumour? And, three, what is the mechanistic basis of cancer cells’ resilience or resistance to our best therapeutic tools?

An evolutionary perspective fits the bill. It provides a framework or context that can make sense of the details. Dobzhansky surely had it right in 1973 when he opined that ‘Nothing in biology makes sense except in the light of evolution.’ Although I doubt if he had pathology and medicine in mind as well.

In 1976, Peter Nowell, in his landmark review in *Science*, highlighted that cancer clones evolve or change over time, and that this has relevance to treatment. Embed that concept within a Darwinian ecosystem context, and empower it with genomic insights, and we have the essence of cancer clone development, as most practitioners of cancer research and oncology now recognise. It's taken a while.

But it is much more than that. I have argued that by focusing on proximate causal factors (important though they may be from the perspective of prevention) we miss a whole dimension of risk. Cancer risk is inherent to the evolutionary ‘design’ of genome mutability, stem cells, multicellularity, and our prior (beneficial) adaptations; for example with skin pigmentation and hormonal cycles. For me, causal exposures only make sense in that context.

And, finally, the resilience of advanced cancer to therapeutic assault is perhaps what you should expect of a semi-autonomous and robust cellular parasite. They can call on survival tactics for which there is a billion-year memory – playing by numbers with rare escapees fuelled by the lottery of random mutation, exploiting signal network plasticity or hunkering down in a dormant state. It's survival of the fittest.

I started my scientific career in the 1960s learning evolutionary biology from John Maynard Smith and others, so I was primed, and may be biased, from that point on. But somehow the answer now just seems so dammed obvious.

## How are new technologies enabling evolutionary studies?

There is no substitute for creative ideas in science but, unquestionably, technological innovations greatly fuel and accelerate progress. They are enabling.

Evolutionary studies in cancer to date, especially on cancer clone evolution, have benefited in particular from single-cell analytical methods: monoclonal antibody-based immune-phenotyping, gene expression signatures, genotypes and lineage-tracking *in vivo*. These tools have revealed remarkably complex and dynamic phylogenetic architectures of cancer cell clones and their variegated genetics.

Tracking clonal evolution in real time via sensitive serial screening of cell-free DNA in plasma now offers the prospect of monitoring the emergence of recurrent or resistant clones, enabling prompt intervention. This circumvents some of the problems linked to the complex topography of subclonal distribution in tissues and the inherent selectivity or bias of biopsies.

## Where do you feel cancer evolutionary biology will have the biggest impact?

The only thing that we can reliably predict about the future is that it will happen. I certainly hope that an evolutionary perspective will strongly endorse the importance and potential impact of prevention and early intervention in cancer. The epidemiological evidence linking most common cancers to potentially avoidable or modifiable lifestyle factors is strong. Early tumours in most, although perhaps not all cases, will be less resilient to challenge. A caveat is that we need to be smarter at distinguishing or predicting bad from benign players.

The biggest challenge is how best to thwart the evolutionary resilience of cancers that present late in the clinic, for example those of pancreas, lung, brain, and ovary. Evolutionary principles and methods are likely to be important, and there is much that could be learned or borrowed from other fields – antibiotic resistance in bacteria and drug resistance in malaria, tuberculosis, and HIV.

Some promising ideas are currently being explored including therapeutic combinations, coopting the versatility of recognition of the immune system into an arms race in cancer. A new and exciting prospect is evolutionary steering. Studies in simpler microbial systems suggest that it may be possible to design scheduling of drug combinations to steer or push cancer clone evolution into a more benign cul-de-sac.

## What are the most exciting questions in cancer evolution?

Once you embrace an evolutionary perspective, a host of exciting questions are opened up. For me, one of the most interesting is whether it is possible to both predict in advance and then modify cancer clone evolutionary trajectories.

I subscribe to the view that cells with self-renewal, or stem cell-like potential, are the cellular drivers of cancer evolutionary progression, metastasis, and recurrence or relapse. The necessary caveat is that this is not an inherent or stable state. I am excited by the prospect of learning more about how the self-renewal option operates and is regulated. Personalised, genome-guided therapeutics are a real if challenging prospect, but isn’t there an opportunity also for a more generic approach tackling cancer via the essential self-renewal bottleneck?

## What other issues or challenges remain unresolved in the drive to understand and exploit the evolutionary biology of cancer?

A whiff here of Rumsfeld's unknown unknowns? We do have some important known unknowns. We still remain ignorant of some of the crucial biology of cancer cells and are probably underestimating complexity. I am somewhat wary of a very gene-centric approach. It would be interesting and potentially of practical value if we had more insight into the ecosystem pressures that select for improved fitness of cancer cells. But then to understand cellular fitness, we also need to have a better grasp of how inherited gene variants and acquired mutations operate collectively or epistatically in a network providing the phenotypic substrates for selection.

Despite being a biologist rather than a clinician, I have always been a strong advocate of ‘real’ or patient-based studies. Nevertheless, I suspect that further validation and understanding of the ecology of cancer will require better models of the disease *in vivo* and in more realistic 3D culture systems.

## Are there other areas of medicine where evolutionary thinking has or can make a difference?

Certainly, and most obviously with antibiotic resistance, which has recently been highlighted at the UN as an urgent, worldwide problem. Evolutionary or Darwinian medicine is a relatively new branch of biomedical sciences that seeks to apply evolutionary principles to all areas of medicine including chronic diseases of modern societies – diabetes, obesity, and neurodegenerative conditions.

Perhaps the clearest example, other than cancer, of evolutionary ideas being highly pertinent to medicine is with infectious disease. Here we witness, not dissimilarly to cancer, an arms race between rapidly-evolving pathogenic species and the immune surveillance system. Vaccination can tip the balance in our favor, but high mutation rates and fast replication are winning cards for the bugs.

## Overall, are you optimistic that evolutionary studies of cancer will be beneficial?

One must be an optimist to survive and thrive in science. But there are real and tangible reasons for being optimistic that an evolutionary perspective on cancer will pay rich dividends; not least it's logical coherence, technological underpinning, and the superb cadre of young scientists now active in the field.

